# Control elements targeting *Tgfb3* expression to the palatal epithelium are located intergenically and in introns of the upstream *Ift43* gene

**DOI:** 10.3389/fphys.2014.00258

**Published:** 2014-07-07

**Authors:** Jamie Lane, Kenji Yumoto, Justin Pisano, Mohamad Azhar, Penny S. Thomas, Vesa Kaartinen

**Affiliations:** ^1^Department of Biologic and Materials Sciences, University of Michigan School of DentistryAnn Arbor, MI, USA; ^2^Department of Pediatrics, Indiana University School of MedicineIndianapolis, IN, USA

**Keywords:** growth factors, craniofacial development, mouse, gene expression, transforming growth factor beta

## Abstract

*Tgfb3* is strongly and specifically expressed in the epithelial tips of pre-fusion palatal shelves where it plays a critical non-redundant role in palatal fusion in both medial edge epithelial (MEE) cells and in a thin layer of flattened peridermal cells that covers the MEE. It is not known how *Tgfb3* expression is regulated in these specific cell types. Using comparative genomics and transgenic reporter assays, we have identified cis-regulatory elements that could control *Tgfb3* expression during palatogenesis. Our results show that a 61-kb genomic fragment encompassing the *Tgfb3* gene drives remarkably specific reporter expression in the MEE and adjacent periderm. Within this fragment, we identified two small, non-coding, evolutionarily conserved regions in intron 2 of the neighboring *Ift43* gene, and a larger region in the intervening sequence between the *Ift43* and *Tgfb3* genes, each of which could target reporter activity to the tips of pre-fusion/fusing palatal shelves. Identification of the cis-regulatory sequences controlling spatio-temporal *Tgfb3* expression in palatal shelves is a key step toward understanding upstream regulation of *Tgfb3* expression during palatogenesis and should enable the development of improved tools to investigate palatal epithelial fusion.

## Introduction

Failure of palatogenesis (palate formation) results in cleft palate, which is one of the most common congenital birth defects in humans. In mice, palatogenesis starts around embryonic day 11.5 (E11.5) when bilateral outgrowths of the maxillary process called palatal shelves start to grow down vertically on each side of the tongue. Co-ordinated growth of palatal shelves themselves, the tongue and the rest of the oral cavity is followed by rapid palatal shelf elevation (~E14) and fusion (~E15) (Bush and Jiang, 2012).

Palatal shelves are composed of the neural crest-derived mesenchyme covered by epithelial cells (Bush and Jiang, 2012). Before palatal fusion, the epithelial layer is composed of a basal layer of cuboidal medial edge epithelial (MEE) cells and an apical periderm layer of flattened cells. This periderm layer is shed from the tips of the apposed elevated palatal shelves just before they form contact with one other, allowing adhesion and intercalation of the underlying MEE cells (Yoshida et al., 2012) to form a midline epithelial seam. Epithelial cells in this seam are subsequently lost and the underlying basement membrane degraded resulting in palatal mesenchymal confluence (Gritli-Linde, 2007).

Several studies have demonstrated that signaling triggered by transforming growth factor-β3 (TGF-β3) plays a critical role in palatal epithelial fusion. *Tgfb3* is strongly and specifically expressed in MEE cells (Fitzpatrick et al., 1990; Pelton et al., 1990; Millan et al., 1991), and mice lacking *Tgfb3* display 100% penetrant cleft secondary palate (Kaartinen et al., 1995; Proetzel et al., 1995), which results from defects in TGF-β3-induced palatal MEE differentiation and/or apoptosis (Kaartinen et al., 1997; Taya et al., 1999; Ahmed et al., 2007; Iwata et al., 2013). Results of a recent study also suggest that TGF-β3 is required for peridermal desquamation (Wu et al., 2013). Mutations in the human *TGFB3* have been linked to cleft palate (Lidral et al., 1998; Carinci et al., 2007), and a recent report described a disease-causing mutation in the coding region of *TGFB3* in patients showing abnormalities in palate and muscle development (Rienhoff et al., 2013).

A commonly used approach to study complex developmental processes has been to manipulate gene function in mouse models using the *Cre-lox* system (Rajewsky et al., 1996). In the context of palatogenesis, an epithelium-specific *keratin14-Cre (K14-Cre)* driver line (Andl et al., 2004) has been frequently used, since it recombines with a very high efficiency in the MEE (Dudas et al., 2006; Xu et al., 2006). Yet abrogation of the *Tgfbr1* gene encoding the TGF-β type I receptor (Dudas et al., 2006) or *Tgfb3* in the palatal epithelium (this study) resulted in a significantly milder palatal phenotype than systemic deletion of the *Tgfb3* gene encoding the TGF-β3 ligand (Kaartinen et al., 1995; Proetzel et al., 1995). Here we show that this phenotypic difference is likely caused by an inability of the *K14-Cre* driver to recombine in peridermal cells.

To better understand how gene expression is specifically directed in the pre-fusion MEE and overlying peridermal cells, we decided to identify control elements responsible for palate-specific *Tgfb3* expression. We surveyed more than 400 kilobases (kb) of mouse genomic DNA sequences on mouse chromosome 12, and identified a 61-kb fragment around the *Tgfb3* gene that directs reporter expression specifically in the MEE and adjacent periderm. Using transient transgenic approaches, we identified three smaller cis-regulatory regions: one in the proximal intergenic region and two in intron 2 of the upstream *Ift43* gene. These more distal elements may function as “shadow” enhancers assuring robust and reliable control of *Tgfb3* expression in the MEE and adjacent periderm.

## Experimental procedures

### Animal care

This study was carried out in accordance with the recommendations of the Guide for the Care and Use of Laboratory Animals of the National Institutes of Health. All the experiments involving animals described in this study were approved by the Animal Care and Use Committee of the University of Michigan-Ann Arbor (protocol number: PRO00004320).

#### BACs and BAC recombineering

Mouse *BACs RP23-76M13 (=5′BAC) and RP24-299H18 (=3′BAC)* were obtained from Children's Hospital Oakland Research Institute (http://bacpac.chori.org) (see **Figure 2A**). Their identity was verified using a standard restriction mapping technique (data not shown).

#### Insertion of the SA-lacZ-PA cassette into exon1 of the 5′ BAC RP23-76M13 and the 3′ BAC RP24-299H18 (see **Figure 2A**)

Targeting arms were generated by PCR using BAC *RP23-76M13* as a template and the following primers:

Tgfb3-L1: 5′-TCCTAGCTCTACCCAGCACACG-3′
Tgfb3H3Xh-L2: 5′-AAGCTTCTCGAGTGTGTGAGCCCAGGAACGAG-3′
Tgfb3XhH3-R1: 5′-CTCGAGAAGCTTGCAAAGGGCTCTGGTAGTCCTG-3′
Tgfb3R2: 5′-TGATAGGGGACGTGGGTCATC-3′

*pNASSβ* (*SA-lacZ-PA* cassette) was inserted into exon 1 of the BACs *RP23-76M13* and *RP24-299H18* using standard BAC recombineering techniques (Warming et al., 2005). *Neo 452* (a *loxP-Neo-PA-loxP* cassette) was added to the generated BAC to enable selection with kanamycin. Integrity of the recombineered BACs was confirmed by PCR after amplification.

#### Preparation of the 61-kb and 28-kb BACs

The *61-kb BAC:* A 128-kb 3′fragment from the recombineered BAC *RP24-299H18* was deleted in two steps. First, a targeting vector to replace the large 3′fragment with *pGalK* was generated by using the primers:

*3′del-F*: 5′-TGACAGATATAGGCAGTGTAAGAACTCGCCATTAGCGGGAGGCGCCATCAGTGCCCCCTTCTGAATTCTACCTGTTGACAATTAATCATCGGCA-3′
3′del-R: 5′-CTTTTCCCCTTGAGATAAGGCCTCTCATTGAACCTGAAACTTACTTTGATTGGGCTGGCTTCAGCACTGTCCTGCTCCTT-3′

After successful recombineering, a targeting vector to delete the *pGalK* selection marker was generated by PCR using the following primers:

3′del-pGalK-F: 5′-AGAACTCGCCATTAGCGGGAGGCGCCATCAGTGCCCCCTTCTGAATTCTAACAAAGTCTATACAGTTCCTCACCCTCTGGGAAAAGTAAGTGCTCAAAAC-3′
*3′del-pGalK-R:* 5′-GTTTTGAGCACTTACTTTTCCCAGAGGGTGAGGAACTGTATAGACTTTGTTAGAATTCAGAACGGGGGCACTGATGGCGCCTCCCGCTAATGGCGAGTTCT-3′

The targeting vector was deleted as described (Warming et al., 2005).

The *28 kb BAC:* A 33-kb 5′ fragment was deleted from the 5′end of the 61-kb BAC as outlined above. Primers to generate the targeting vector were:

*5′3′del-F:* 5′-TGACCAGGGAGAGGGGCTGTTATGAGGTACTGGGCATCCTGATGGGATGAGAGAACATTCTCCTGTTGACAATTAATCATCGGCA-3′
*5′3′del-R:* 5′-GGGCAATGGAGATGTCAAACACGGGCTGCCTAATCTGGAAAGGCATTATTTTAACTTGTATCAGCACTGTCCTGCTCCTT-3′

The targeting vector to delete the *pGalK* selection marker was generated by PCR and the following primers:

*5′3′del-pGalK-F:* 5′-AGGGGCTGTTATGAGGTACTGGGCATCCTGATGGGATGAGAGAACATTCTTACAAGTTAAAATAATGCCTTTCCAGATTAGGCAGCCCGTGTTTGACATC3′
*5′3′del-pGalK-R:* 5′-GATGTCAAACACGGGCTGCCTAATCTGGAAAGGCATTATTTTAACTTGTAAGAATGTTCTCTCATCCCATCAGGATGCCCAGTACCTCATAACAGCCCCT-3′

BAC DNAs were purified for microinjections using Nucleobond AX alkaline lysis protocol according to the manufacturer's instructions (Clontech).

### Preparation of smaller reporter constructs

The *2xcHS4-hsp68-lacZ-PA-2xcHS4* vector was generated by replacing a *Sac*II-*Sac*I fragment from the *pUbC-SH-Gm-4xcHS* plasmid (kindly provided by R. Behringer) with the *hsp68-lacZ-PA* cassette. A unique *Not*I site just upstream of the *hsp68* minimal promoter was generated by using the Quikchange-II site-directed mutagenesis kit (Agilent). Regions of interest were PCR-amplified using SuperMix High Fidelity polymerase (Invitrogen) (primer sequences shown in Table 1), and the generated fragments inserted into the *Not*I site using the In-Fusion HD cloning kit (Clontech). Plasmid DNAs were purified using endonuclease-free Maxi-Prep columns (Qiagen) and the purified DNAs were linearized by *Sal*I for microinjection.

**Table 1:** **Primer sequences used for In-Fusion cloning**.

**Fragment**	**Forward primer**	**Reverse primer**
−(6.1–0.8)	TTGGCGCCTCCCGCGGCCGCgatgagcccggcgtcccatctt	GTTTGGATGTTCGCGGCCGCcctttctaagaggcctggttctgg
−(6.1–3.7)	TTGGCGCCTCCCGCGGCCGCgatgagcccggcgtcccatctt	GTTTGGATGTTCGCGGCCGCtctctgagaagctgggagtctg
−(3.7–0.8)	TTGGCGCCTCCCGCGGCCGCttgaatcatttgagaagtgagttt	GTTTGGATGTTCGCGGCCGCcctttctaagaggcctggttctgg
−(13.7–6.1)	TTGGCGCCTCCCGCGGCCGCggatccttctctgtaaagtagac	GTTTGGATGTTCGCGGCCGCgtcgactcaggctgagaatt
−(13.7–9.7)	TTGGCGCCTCCCGCGGCCGgatccttctctgtaaagtagac	GTTTGGATGTTCGCGGCCGCgtgctgcgagccaactgagcc
−(9.7–6.1)	TTGGCGCCTCCCGCGGCCGCcatcaggttagctggaac	GTTTGGATGTTCGCGGCCGCgtcgactcaggctgagaatt
−(7.9–7.6)	TTGGCGCCTCCCGCGGCCGCggcaagccctgtgtctccct	GTTTGGATGTTCGCGGCCGCcccccctggaaacagggtgt
−(7.4–6.6)	TTGGCGCCTCCCGCGGCCGCcacacacacccctgcacaac	GTTTGGATGTTCGCGGCCGCaggcactgggatcaggc
−(13.0–12.5)	TTGGCGCCTCCCGCGGCCGgatggagccgctgattctga	GTTTGGATGTTCGCGGCCGCggggagcagggttggaatcc
−(26.9–24.0)	TTGGCGCCTCCCGCGGCCGCagaccaaggtctgcaagt	GTTTGGATGTTCGCGGCCGCggaactaacacttgtcctg

Capital letters indicate the sequences that are homologous to the vector.

### Alignment of orthologous sequences and identification of putative binding motifs

Multi-species sequence comparisons around the *Tgfb3* gene were performed using the UCSC genome browser (http://genome.ucsc.edu) and VISTA tools for Comparative Genomics (http://genome.lbl.gov/vista) using the global pair-wise and multiple alignment (LAGAN) program. The threshold used for evolutionary conservation was 70% sequence similarity within 100 bp region of DNA sequence. Predicted transcription factor binding sites were identified by using RankVISTA and TRANSFAC matrices.

### Generation of transgenic mouse lines and transient transgenic mouse embryos

The transgenic mouse lines and transient transgenics were generated in the Transgenic Animal Model Core facility at the University of Michigan—Ann Arbor.

### Other mouse lines used in this study

We generated epithelium-specific *Tgfb3* mutants by crossing mice heterozygous for the floxed *Tgfb3* allele *(Tgfb3^FXWT^)* (Doetschman et al., 2012) and carrying the epithelial *K14-Cre* driver (Andl et al., 2004) with homozygous floxed *Tgfb3 (Tgfb3^FXFX^)* mice. *R26R-YFP* reporter mice were obtained from the Jackson Laboratories, and generation of *Tgfb3-Cre* mice has been previously described (Yang et al., 2008).

### X-Gal staining

To detect expression of β-galactosidase encoded by the *lacZ* reporter gene, embryos were collected, washed and fixed in freshly prepared 4% para-formaldehyde-0.5% glutaraldehyde for 20 min, washed 3 × 20 min in the detergent wash solution and stained from 4 h to overnight in X-Gal staining solution as described (Behringer et al., 2003). The stained samples were examined using a Leica MZ95 dissecting microscope and photographed using an Olympus DP71 camera and DP controller and manager software. Selected samples were processed for paraffin embedding using Histoclear, sectioned, rehydrated and mounted in Immumount (Fisher) or couterstained with eosin or Nuclear Fast Red and mounted in DPX.

### Histology and immunohistochemistry

For paraffin embedding, embryos were harvested and fixed in 4% para-formaldehyde for 24 h at +4°C, washed, dehydrated and embedded in Leica Histowax. Sections (7 μm) were stained with hematoxylin and eosin using standard protocols. For immunohistochemistry, the paraformaldehyde fixed samples were allowed to sink in sterile 10% sucrose in PBS, then in 7% gelatin/15% sucrose in PBS, oriented and embedded in fresh 7% gelatin/15% sucrose in PBS on ice, then dry ice, and stored at −80°C. Cryosections (10 μm) were cut and stored at −80°C. The sections were stained with αSSEA-1 (MC-480 from DSHB) and αGFP (A11122 from Life Technology) antibodies, which were detected by Alexafluor-594 and Alexafluor-488 secondary antibodies (Invitrogen) respectively. The stained sections were mounted with Vectashield mounting medium containing DAPI (Vector Labs Inc). Sections were viewed using an Olympus BX51 microscope and documented using an Olympus DP71 digital camera as described above.

## Results

### Palatal peridermal cells are not recombined in a commonly used *K14-Cre* mouse line

**Figure 1:**
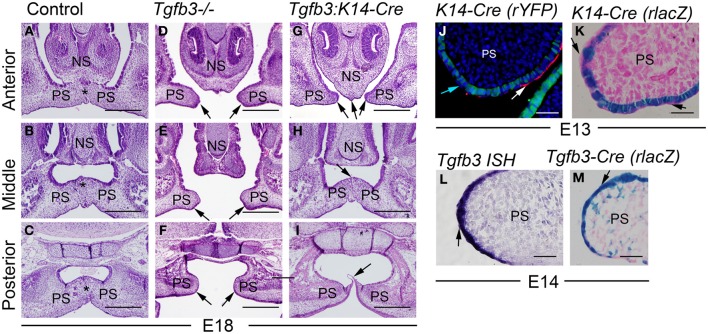
**Milder palatal phenotype of epithelium-specific *Tgfb3:K14-Cre* mutants than that of *Tgfb3* null mutants results from an inability of *K14-Cre* to recombine in peridermal cells. (A–C)** control; **(D–F)** *Tgfb3^-/-^* mutant; **(G–I)**, *Tgfb3:K14-Cre* (**A–I**, frontal orientation; all at E18). **(A,D,G)** on the level of the nasal septum (anterior); **(B,E,H)**, mid-eye level (middle); **(C,F,I)** on the level of soft palate (posterior). Asterisks in **(A–C)** indicate confluent midline mesenchyme, black arrows in **(D,E)** point to unfused palatal shelves, black arrows in **(G–I)** point to unfused elements of the anterior palate **(G)**, a persistent epithelial seam in the mid-palate **(H)**, and an epithelial bridge in the posterior soft palate **(I)**. **(J)** a frontal palatal section of a *K14-Cre:R26R-YFP* embryo at E13; double immuno-fluorescence staining to detect YFP-positive recombined cells (green) and an SSEA1-positive subset of non-recombined peridermal cells (red, white arrow). Light blue arrow points to the DAPI-positive nucleus of a peridermal cell that is SSEA-1-negative and has not been recombined by *K14-Cre*. **(K)** A frontal palatal section of a X-Gal-stained *K14-Cre:R26R-lacZ* embryo at E13, counterstaining with eosin. Black arrows point to apical peridermal cells that were not recombined with *K14-Cre.* **(L)** *In situ* hybridization for *Tgfb3* at E14 (palatal frontal section). Black arrow points to a positively staining flattened cell with peridermal appearance. **(M)** A frontal palatal section of X-Gal-stained *Tgfb3-Cre:R26R-lacZ* embryo, counterstained with eosin. Black arrow points to an X-Gal-positive flattened cell with peridermal appearance. PS, palatal shelf; NS, nasal septum. Scale bars in **(A–I)** 200 μm; **(J–M)** 50 μm.

Comparison of the palatal phenotypes of global *Tgfb3* knockout mice (*Tgfb3^−/−^*) and epithelium-specific *Tgfb3 (Tgfb3:K14-Cre)* mice revealed that, despite the efficient recombination in the MEE, the germline mutants consistently displayed a more severe phenotype than the tissue-specific mutants (Figures 1A–I): *Tgfb3^−/−^* mice had a complete cleft of the secondary palate (Kaartinen et al., 1995; Proetzel et al., 1995), while *Tgfb3:K14-Cre* mice had a cleft anteriorly, but superficial or complete fusion in the mid-palate, and an aberrant posterior epithelial bridge. Since the *Tgfb3:K14-Cre* palatal phenotype was practically identical to that observed in the epithelium-specific TGF-β receptor mutants (both *Tgfbr1:K14-Cre* and *Tgfbr2:K14-Cre*) (Dudas et al., 2006; Xu et al., 2006), we wondered whether this milder palatal phenotype was caused by an inability of the *K14-Cre* driver line (Andl et al., 2004) to induce recombination in peridermal cells. To address this question we harvested tissues from *K14-Cre, R26R-YFP* reporter embryos at E13.5, and assessed the Cre-induced recombination in MEE and peridermal cells (Figures 1J,K). Our results showed that while the MEE was efficiently recombined, we could not detect reporter expression in the adjacent periderm. In contrast, *Tgfb3* was strongly and specifically expressed both in the periderm and underlying MEE as demonstrated by both *in situ* hybridization, and *R26R-lacZ* reporter expression in the *Tgfb3-Cre^KI^* mouse line (Yang et al., 2008) (Figures 1L,M).

### Survey of the *Tgfb3* cis-regulatory function using recombinant reporter BACs

**Figure 2:**
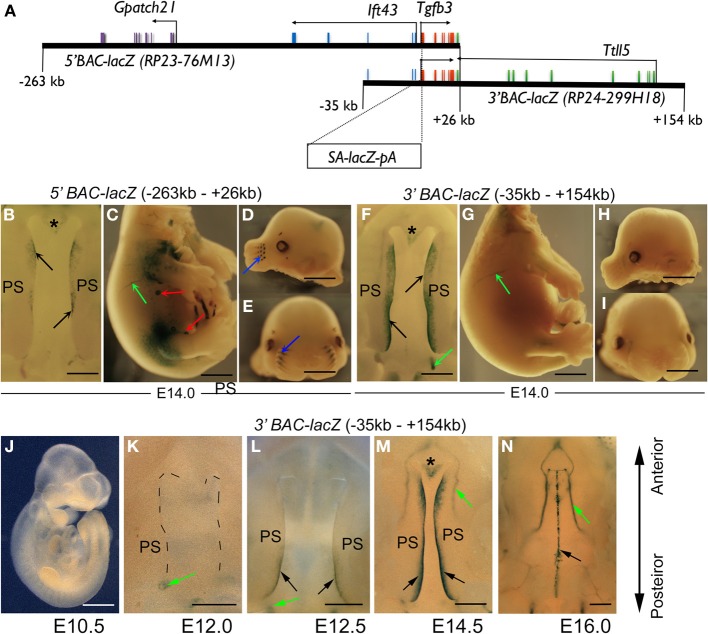
**Recombinant reporter BACs used to detect *Tgfb3* regulatory regions. (A)** Schematic representation of *Tgfb3*-containing *lacZ* BAC clones. Exons of the *Tgfb3* gene are shown as red vertical bars and exons of *Ttll5*, *Ift43*, and *Gpatch21* genes are shown as green, blue and purple vertical bars, respectively. Horizontal black arrows show the direction of transcription of each gene. **(B–E)** *5′ lacZ* BAC transgenic embryos showing β-gal reporter activity (blue staining) in tips of palatal shelves (**B**, black arrows), nasal septum (**B**, asterisk, at anterior end of palate), blood vessels (**C**, green arrow), mammary placodes (**C**, red arrows) and in whisker follicles (**D,E**, blue arrows). **(F–I)**, 3′ *lacZ* BAC transgenic embryos showing β-gal reporter activity (blue staining) in tips of palatal shelves (**F**, black arrows), nasal septum (**F**, asterisk), blood vessels (**F,G**, green arrows). **(B,F)** roof of mouth at E14, inferior view; **(C,G)** torso at E14, right lateral view, **(D,H)** head (mandible removed), left lateral view; **(E,I)** head (mandible removed), frontal view. **(J–N)** Reporter activity in *3′ lacZ*-BAC transgenic embryos between embryonic days 10.5 and 16.0. Black arrows point to expression in tips of pre-fusion palatal shelves **(K,L)** and midline seam **(N)**; green arrows point to positively staining blood vessels **(K–N)**; asterisk (in **M**) marks the nasal septum PS, palatal shelf. **(B,F,K–N)**, anterior, top; posterior, bottom. Scale bars in **(K)**, 450 μm; **(B,F,J,L–N)**, 500 μm; **(C–E,G–I)**, 1 mm.

Since *Tgfb3* is strongly and specifically expressed in peridermal and MEE cells, we reasoned that identification of cis-regulatory elements controlling palate-specific *Tgfb3* expression would be invaluable for development of new improved genetic tools to examine palatal epithelial fusion *in vivo*. The *Tgfb3* gene, composed of 7 evolutionarily conserved exons, is located on mouse chromosome 12 between the *Ift43* (intraflagellar transporter 43) and *Ttl5* (tubulin tyrosine ligase-like 5) genes, which both lie in the opposite orientation to the *Tgfb3* gene (Figure 2A). The intergenic flanking sequences are remarkably short (3–3.5 kb) but the neighboring genes are not expressed in pre-fusion palatal shelves (**Figures 4A,B**).

To assess large regions upstream and downstream of the *Tgfb3* gene for regulatory elements, we obtained two overlapping BACs. The 5′ BAC *(RP23-76M13)* contained a 289-kb region from −263 to +26 kb [defining *Tgfb3* transcriptional start site (TSS) as 0] which included the *Tgfb3, Ift43*, and *Gpatch2l* genes (Figure 2A). The 3′ BAC (*RP24-299H18*) contained the 189-kb region from −35 to +154 kb which included the *Tgfb3* gene and the *Ttll5* (variant 4) gene (Figure 2A). The sequences of the two BACs overlapped by 61 kb, which includes all the *Tgfb3* exons and some of those of the neighboring genes.

**Figure 3:**
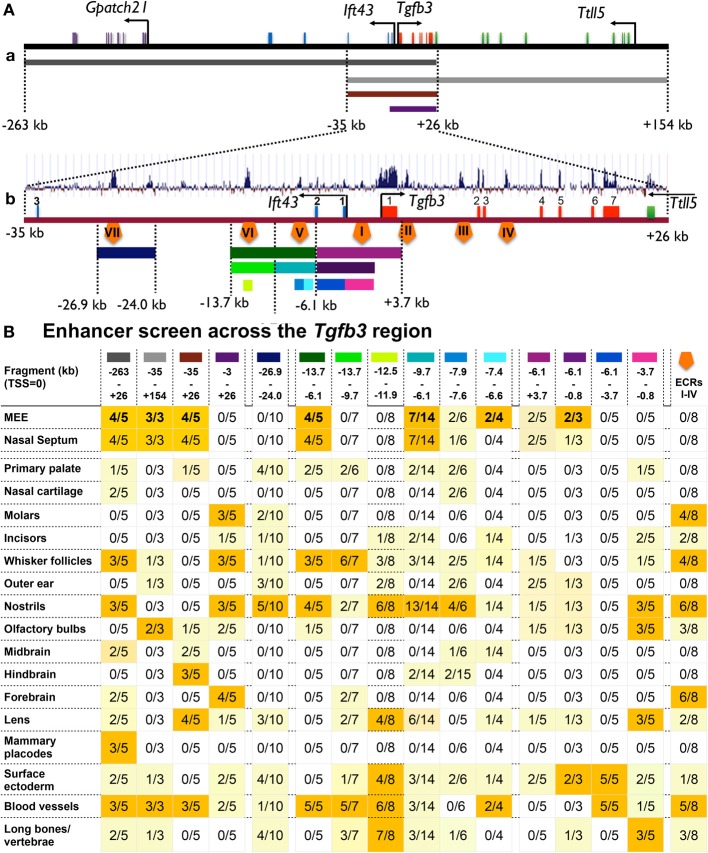
**Enhancer screening across the *Tgfb3* region. (A)** Schematic representation of the regions examined to locate cis-regulatory sequences directing reporter activity to the MEE/periderm cells. **(a)** 417-kb of genomic DNA (black line) including the *Tgfb3* gene (red boxes represent exons); colored lines beneath correspond to the positions of *BAC* sequences used for expression regulation analysis (see **B**, first four columns). **(b)** Schematic representation of the 61-kb region in common between the 5′ and 3′ BACs (brown line) showing the *Tgfb3* gene (exons in red), exons 1–3 of the *Ift43* gene (blue boxes) and exon 16 of the *Ttll5* gene (green box). Graph above shows evolutionary sequence conservation among placental mammals (UCSC genome browser) along this sequence. Orange pentagons indicate positions of ECRs I-VII (see main text); colored lines beneath correspond to the positions of regions used for regional expression regulation analysis (see **B**, fifth column onwards). **(B)** A table summarizing findings of reporter activity driven by the sequences in the regions indicated by colored bars in **(A)** in various tissues of transgenic embryos at E14. Data entries show the number of embryos displaying positive reporter activity in selected tissues (rows) over the total number of *lacZ*-positive embryos for each sequence (identified by a colored bar in each column); dark yellow highlight, 50% or more staining; pale yellow highlight, >0%, <50% staining.

To prepare reporter constructs, we inserted an *SA-lacZ-pA* cassette into *Tgfb3* exon 1 of each BAC using standard recombineering techniques (Warming et al., 2005). These recombinant *lacZ* reporter BACs were used to generate transgenic mice and β-galactosidase activity assessed at E14.0 using X-Gal staining as *Tgfb3* is usually strongly expressed in palatal shelf MEE and nasal septal epithelium around this stage. Both *5′* and *3′ lacZ*-BACs were able to target reporter activity correctly to the palatal midline and nasal septal tissues (Figures 2B,F). The 5′ *lacZ*-BAC transgenic embryos were also stained in mammary placodes, whisker follicles, nostrils, and vasculature (Figures 2C–E), while the 3′*lacZ*-BAC embryos showed additional staining principally in vasculature (Figures 2G–I, 3, which illustrates the positions of all DNA fragments tested for enhancer activity in this study and summarizes expression data). Stable transgenic mouse lines carrying *3′ lacZ*-BAC did not show detectable reporter activity until E12.0–E12.5 (Figure 2J), when staining was seen first in blood vessels and soon afterwards in the tips of the posterior palatal shelves (Figures 2K,L). At E14.5 strong staining occurred along the entire A-P axis of the palatal shelf tips, and continued during and after palatal epithelial fusion when it could still be detected in the degrading midline seam and in vasculature at E16.0 (Figures 2M,N).

**Figure 4:**
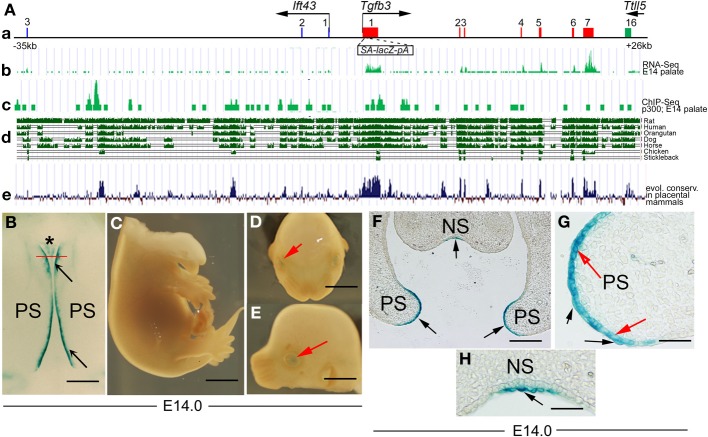
**A 61-kb genomic region including the*Tgfb3* gene targets reporter activity to the MEE and adjacent periderm. (A)** Schematic representation of the 61-kb region (see main text) includes **(a)** the *Tgfb3* gene (exons in red); exons 1–3 of the *Ift43* gene (blue boxes) and exon 16 of the *Ttll5* gene (green box). That *Tgfb3* but neither of its neighbors is actively expressed at E14.5 is shown in **(b)**, RNA-seq profile in mouse palate at E14.5—(FaceBase Enhancer Project; A Visel). Different types of sequence analysis are suggestive of possible enhancer sites: **(c)** p300 Chip-seq profile in mouse palate at E14.5 (ref: FaceBase Enhancer Project; A Visel), **(d)** evolutionary sequence conservation among selected vertebrate species in this sequence (UCSC genome browser) and **(e)** evolutionary sequence conservation among placental mammals (UCSC genome browser). **(B–E)** 61-kb *BAC-lacZ* embryos showing β-gal activity (blue staining) in tips of palatal shelves (**B**, black arrows), nasal septum (**B**, asterisk), and lens (**D,E**, red arrows). **(B)** Roof of mouth at E14, inferior view, anterior on the top; **(C)** torso at E14, right lateral view; **(D)** head (mandible removed), left lateral view; **(E)** head (mandible removed), frontal view. **(F–H)** Frontal sections of an X-Gal stained 61-kb *lacZ*-BAC embryo at the level of nasal septum (indicated by the red line in **B**). **(F)** X-Gal staining (black arrows) at the tips of the pre-fusion palatal shelves (PS) and in the nasal septum (NS) is present in cells of both the basal MEE layer (red arrows in **G**) and the overlying periderm layer (black arrows in **G**), and periderm in the nasal septum (black arrow in **H**). Scale bars in **(B)**, 500 μm; **(C–E)**, 1 mm; **(F)** 200 μm; **(G,H)**, 50 μm.

As the 5′ and 3′ BAC sequences overlapped, and both the 5′ and 3′ *lacZ*-BAC reporters drove expression in the palatal shelf tips, we hypothesized that sequences in the region common to each *BAC* may be responsible. We tested this by making a reporter *lacZ*-BAC containing only this sequence, from −35 to +26 kb (Figures 3A, 4Aa). This 61-kb fragment of mouse genomic DNA consistently drove highly specific reporter expression in the MEE and the adjacent periderm, and in peridermal cells covering the nasal septum where anterior secondary palatal fusion occurs (Figures 4B,F–H). The only other tissue showing detectable though weak X-Gal staining was the lens (Figures 4D,E).

### Noncoding evolutionarily conserved sequences within the *Tgfb3* gene are not responsible for the MEE-specific gene expression

**Figure 5:**
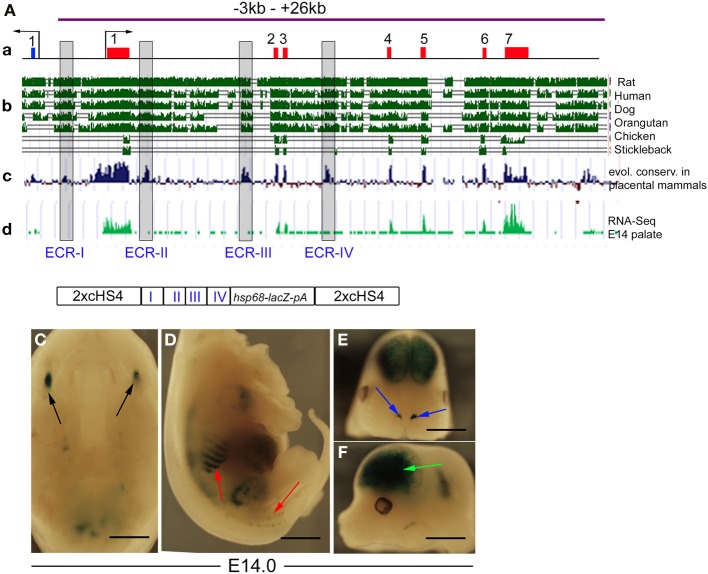
**Evolutionarily conserved regions within the *Tgfb3* gene do not direct reporter expression in palatal shelves. (A)** Schematic representation **(a)** of the *Tgfb3* gene (red boxes depict *Tgfb3* exons 1–7; blue box depicts *Ift43* exon 1; black arrows show the TSSs for *Tgfb3* and *Ift43*) aligned with graphs of evolutionary sequence conservation among selected vertebrate species (**b**, UCSC genome browser), among placental mammals (**c**, UCSC genome browser) and RNA-seq profile in mouse palate at E14.5 (**d**, FaceBase Enhancer Project; A Visel) used to identify non-coding, evolutionarily conserved regions (ECRs I–IV: gray boxes). Purple line indicates the region present in the 29-kb *lacZ*-BAC. **(B)** Schematic presentation of the reporter construct used to generate transgenic embryos shown in **(C–F)**. **(C–F)**, *ECR(1-IV)-hsp68-lacZ* embryos at E14 showing β-gal activity (blue staining) in molars (**C**, black arrows), ribs and lower spine (**D**, red arrows), and forebrain (**F**, green arrow). **(C)** Mouth roof at E14, inferior view, anterior at the top; **(D)** torso at E14, right lateral view, **(E)** head (mandible removed), frontal view; **(F)** head (mandible removed), left lateral view. Scale bars in **(C)**, 700 μm; **(D–F)**, 1 mm.

We analyzed the 61-kb overlapping region using the UCSC Genome Browser (http://genome.ucsc.edu) (Figures 4Ac–e) to identify non-coding evolutionarily conserved regions (ECRs), which are likely to include tissue-specific enhancers and found four: ECR-I (2 kb upstream of TSS), ECR-II and ECR-III (in *Tgfb3* intron 1) and ECR-IV (in *Tgfb3* intron 3) (Figure 5). As these are all highly conserved in placental mammals, which develop a complete secondary palate, but not in avians (or fish), which do not express *Tgfb3* in tips of palatal shelves and do not develop the fused secondary palate (Sun et al., 1998), they were good candidates to regulate palate-specific expression. To test this, the ECRs (I-IV) were PCR-amplified and subcloned upstream of the minimal *hsp68* promoter and *lacZ-PA* reporter, and the resulting ECR-(I-IV)–*hsp68-lacZ-PA* cassette cloned between concatamerized pairs of genomic insulators (cHS4), which have been shown to reduce positional effects of transgenes and alleviate promoter interference (Yahata et al., 2007; Griswold et al., 2011) (Figure 5). Transient transgenic embryos were generated and analyzed for reporter activity at E14.0. While the ECRs were consistently able to target the reporter activity to several tissues (teeth, whisker follicles, nostrils and forebrain), no staining was seen in the MEE (*n* = 15) (Figure 5). We also modified the 61-kb *lacZ*-BAC by deleting sequences from −35 to −3 kb. This 28-kb BAC was also unable to direct *lacZ* reporter expression to the MEE (Figure 3). These data suggest that the sequences from −3.0 to +26 kb, including the entire *Tgfb3* gene and ECRs I-IV, are not responsible for the MEE/ periderm-specific gene expression in mouse embryos during palatogenesis.

### Cis-regulatory elements directing gene expression in the MEE are located in intron 2 of the upstream Ift43 gene

**Figure 6:**
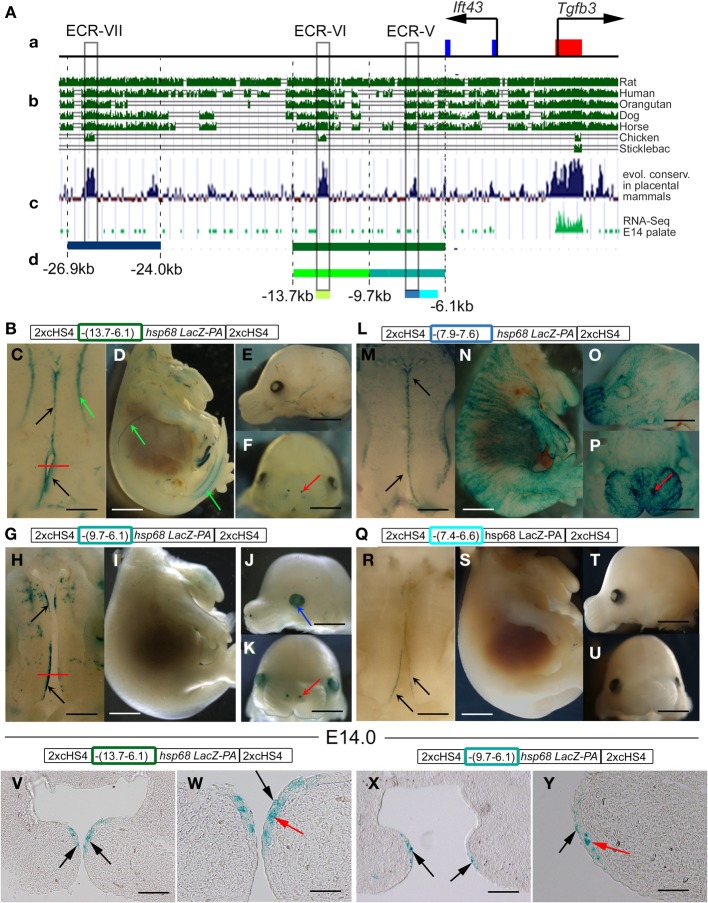
**Cis-regulatory elements targeting reporter activity to the MEE and adjacent periderm are located in intron 2 of the upstream *Ift43* gene. (A)** Schematic representation of a 28-kb sub-region of the 61-kb genomic fragment (**a**, see Figure 4 and the main text) that includes *Tgfb3* exon 1 (red box) and exons 1 and 2 of the *Ift43* gene (blue boxes), aligned with **(b)** evolutionary sequence conservation among selected vertebrate species (UCSC genome browser), and **(c)** among placental mammals (UCSC genome browser), and **(d)** RNA-seq profile in mouse palate at E14.5 (FaceBase Enhancer Project; A Visel) used to identify the positions of non-coding evolutionary conserved regions (ECRs-V, -VI, and -VII: gray boxes). Colored bars below **(d)** correspond to DNA fragments (see also Figure 3) examined by transient transgenic reporter assay in constructs shown schematically above images of the stained embryos generated **(B–Y)**. **(C–F)** Transgenic reporter embryos carrying a 7.6-kb DNA fragment from −13.7 to −6.1 kb (green bar below **(c)** and green rectangle in construct schematic) showing β-gal activity (blue staining) in tips of palatal shelves (**C**, black arrows), blood vessels (**C,D**, green arrows) and nostrils (**F**, red arrow). **(H–K)** Transgenic reporter embryos carrying a 3.6-kb DNA fragment from −9.7 to −6.1 kb (blue-green bar below **(c)** and blue-green rectangle in construct schematic) showing β-gal activity (blue staining) in tips of palatal shelves (**H**, black arrows), lens (**J**, blue arrow) and nostrils (**K**, red arrow). **(M–P)** Transgenic reporter embryos carrying a 0.3-kb DNA fragment (ECR-V) from −7.9 to −7.6 kb [light blue bar below **(c)** and light blue rectangle in construct schematic] showing β-gal activity (blue staining) in tips of palatal shelves (**M**, black arrows), apical ectoderm **(N–P)** and nostrils (**P**, red arrow). **(R–U)** Transgenic reporter embryos carrying a 0.8-kb DNA fragment from −7.4 to −6.6 kb (turquoise bar below **(c)** and rectangle in construct schematic) showing β-gal activity (blue staining) in tips of posterior palatal shelves (**R**, black arrows). **(V–W)** Frontal sections of the X-Gal-stained 7.6-kb fragment transgenic embryo shown in **(C)** at the level of the posterior palate (indicated by the red line in **C**). Staining at the tips of palatal shelves (black arrows in **V**) is in both MEE cells (red arrow in **W**) and periderm cells (black arrow in **W**). **(X,Y)** Frontal sections of the X-Gal-stained 3.6-kb fragment transgenic embryo shown in **(H)** at the level of the posterior palate (indicated by the red line in **H**). Staining at the tips of palatal shelves (black arrows in **X**) is in both MEE cells (red arrow in **Y**) and periderm cells (black arrow in **Y**). Scale bars in **C,H,M,R**, 500 μm; **D,I,N,S,E,F,J,K,O,P,T,U**, 1 mm; **V,X**, 100 μm; **W,Y**, 50 μm.

In addition to the noncoding ECRs I-IV, the 61-kb region from −35 to +26 kb contains three additional highly conserved regions in intron 2 of the *Ift43* gene: ECR-V at position −(7.9 to 7.6)kb, ECR-VI at −(13.0 to 12.5)kb and ECR-VII at −(26.9 to 24.0)kb. To assess these regions for the possible presence of MEE/periderm-specific cis-regulatory elements, we first subcloned the ECRs -V and -VI into the 2xcHS4-*hsp68-lacZ-2xcHS4* vector as a single 7.6-kb fragment (Figure 5A) and ECR-VII as a 2.9-kb fragment (Figure 5A). Analysis of X-Gal-stained transient transgenic embryos at E14 revealed that the region surrounding ECR-VII targeted the reporter activity to vascular and skeletal structures, but did not direct reporter activity in the MEE (Figure 3, and data not shown). In contrast, the 7.6-kb region containing both ECRs -V and -VI was able to drive expression not only in the MEE/periderm with high efficiency (7/14) (Figures 3B, 6B,C,V,W), but also in the vasculature (including palatal vessels), nostrils and whisker follicles (Figures 6C–F). Within this region, ECR-V is conserved only in mammals but ECR-VI is conserved in both mammals and avians suggesting that ECR-V would be more likely to contain palate-specific control elements. Indeed, this was the case, since the sequences between −9.6 and −6.1 kb including ECR-V targeted reporter activity to the MEE/periderm (Figure 3 and Figures 6G–K,X,Y), while ECR-VI and surrounding sequences (from −13.7 to −9.7 kb) did not (Figure 3 and data not shown). To narrow down the regions within −9.7 and −6.1 kb that contained putative cis-regulatory modules, we next examined a 0.3-kb fragment that encompassed the highly conserved ECR-V (from −7.9 to −7.6 kb), and an adjacent conserved 0.8-kb region from −7.4 to −6.6 kb (Figure 6A). Each region drove reporter expression in the tips of palatal shelves but relatively weakly (Figures 3, 6L–U) and with far less specifically than the larger (−13.7 to −6.1 kb) fragment. These results suggest that the 3.5-kb region in *Ift43* intron 2 contains two or more cis-regulatory elements independently able to direct the reporter activity in the MEE and adjacent periderm, but they are needed in combination to drive expression efficiently.

### An additional cis-regulatory region is located in a 5.3-kb fragment immediately upstream of *Tgfb3* exon 1

**Figure 7:**
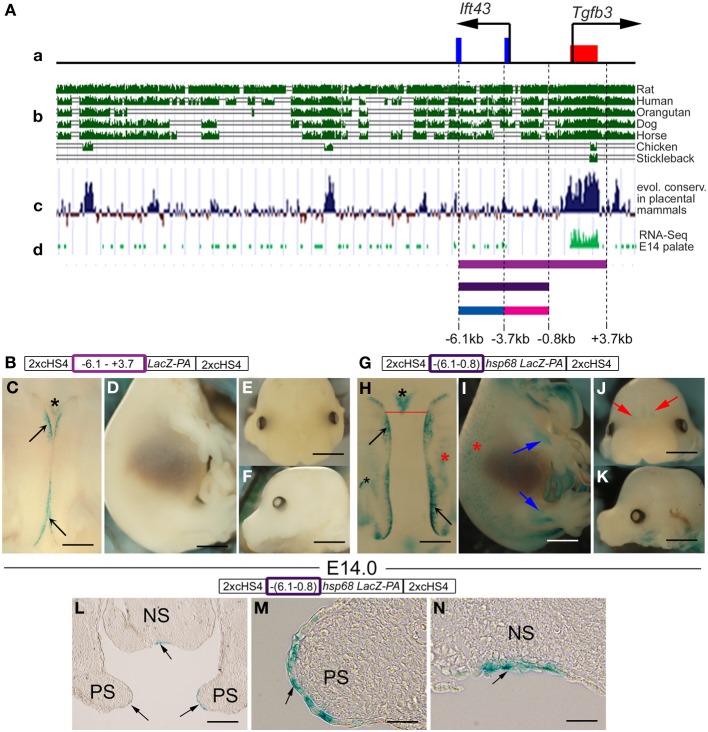
**A putative proximal enhancer directing palatal expression lies in a 5.3-kb region upstream of the *Tgfb3* gene. (A)** Schematic representation **(a)** of a 28-kb region upstream of *Tgfb3* exon 1 (red box) including *Ift43* exons 1 and 2 (blue boxes) aligned with **(b)** evolutionary conservation among selected vertebrate species (ucsc genome browser), **(c)** evolutionary conservation among the placental mammals (ucsc genome browser), **(d)** RNA-seq profile in mouse palate at E14.5. Colored bars below **(d)** correspond to DNA fragments examined using transient transgenic reporter assays **(B–N)**. **(B,G)** Schematic presentations of reporter constructs used to generate transgenic embryos [colored rectangles correspond to colored bars shown above **(a)**]. **(C–F)** Transgenic reporter embryo carrying the 9.8-kb DNA fragment from −6.1 to +3.7 kb (magenta bar above **(a)** and magenta rectangle in **B**) showing β-gal activity (blue staining) in tips of palatal shelves (**C**, black arrows) and in the nasal septum (asterisk in **C**). No staining was seen in the torso **(D)** or head **(E,F)**. **(H–K)** Transgenic reporter embryo carrying the 5.3-kb DNA fragment from −6.1 to −0.8 kb [dark purple bar above **(a)** and rectangle in **G**] showing β-gal activity (blue staining) in tips of palatal shelves (**H**, black arrows), in nasal septum (asterisk in **H**), in the ectoderm (red asterisks in **H,I**) and in skeletal structures (**I**, blue arrows) and olfactory bulbs (**J**, red arrows). **(L–N)** Frontal sections of the X-Gal-stained 5.3-kb transgenic embryo shown in **(H)** at the level of the nasal septum (indicated by the red line in **H**). Staining can be seen at the tips of palatal shelves (black arrows in **L,M**) and in periderm cells of the nasal septum (black arrow in **N**). Scale bars in **(C,H)**, 500 μm; **(D,I,E,F,J,K)**, 1 mm; **(L)**, 200 μm; **(M,N)**, 50 μm.

Since the overall conservation of the intergenic region between the *Ift43* and *Tgfb3* genes is relatively high, we examined whether this region could also contribute to MEE/periderm-specific expression (Figure 7A). First we cloned the 9.8-kb region from −6.1 to +3.7 kb (i.e., *Ift43* intron 1, *Ift43* exon 1, intervening sequences between the *Ift43* and *Tgfb3* genes and *Tgfb3* exon 1) between concatamerized pairs of *cHS4* insulators, and inserted a *lacZ-PA* cassette in frame into the *Tgfb3* exon 1 (Figure 7B). This construct, driven by the endogenous *Tgfb3* promoter, directed reporter activity specifically in the palatal midline region in transient transgenic embryos, although with a relatively low frequency (2/5) (Figures 3B, 7B–F). To further define the important region within this 9.8 kb fragment, we subcloned the 5.3-kb region from −6.1 to −0.8 kb into the 2xcHS4-*hsp68-lacZ-2xcHS4* vector, as it lacks the endogenous *Tgfb3* promoter (Figure 7G). Two of the three resulting transgenic embryos showed reporter activity in the palatal midline (Figure 7H) though also in several other tissues (Figures 3, 7I–N), suggesting that the shorter sequence lacked elements necessary for highly regionally specific regulation. Intron 1 of *Ift43* (from −6.1 to −3.7 kb) alone, or the intervening sequence between *Ift43* and *Tgfb3* (from −3.7 to −0.8 kb), did not direct β–galactosidase activity in the tips of palatal shelves (*n* = 5 in each case) (Figure 3B and data not shown). These data imply that a fragment from *Ift43* intron 1 to *Tgfb3* exon 1 contains a putative proximal MEE/periderm enhancer, which is dependent on DNA sequences separately located in smaller fragments.

## Discussion

Conditional Cre drivers are an invaluable tool for investigating the roles and timing of gene expression in processes involving several cell types such as palatogenesis. Key to this is knowledge of their recombination patterns and efficiency. Here we have shown that *K14*-Cre recombines efficiently in palatal medial edge epithelium (MEE) but it is not expressed in the overlying palatal periderm. This could explain the phenotypic differences between the germline *Tgfb3* mutants (in which no *Tgfb3* is expressed by MEE or periderm, periderm is inadequately shed and a complete cleft of the secondary palate occurs) and the epithelium-specific *Tgfb3:K14-Cre* mutants (in which genotypically normal periderm itself may be providing sufficient TGF-β3 signaling for some shedding, and thus a milder phenotype occurs). This proposed role for peridermal *Tgfb3* expression, and known peridermal responsiveness to TGF-β3-triggered signaling (Wu et al., 2013) combined with an inability of *K14-Cre* to recombine in peridermal cells could also explain why periderm behaves normally in *Tgfbr2:K14-Cre* mutants (Iwata et al., 2013). In contrast with these TGF-β3 signaling, *K14*-Cre conditional knock-outs, epithelium-specific β-catenin mutants (*Ctnn1b:K14-Cre*) lose *Tgfb3* expression in tips of palatal shelves but still develop total cleft of the secondary palate (He et al., 2011), raising the intriguing possibility that canonical Wnt signaling is specifically required in MEE for *Tgfb3* expression to occur in both the MEE and adjacent periderm. To test these and related hypotheses other conditional Cre drivers are required: to recombine only in palatal periderm, and to recombine in both MEE and the overlying periderm. It is not clear whether all other Cre-drivers currently used to delete genes in the palatal epithelium recombine in periderm as well, and many have additional limitations: *Pitx2-Cre* recombines predominantly in the posterior palatal epithelium (Xiong et al., 2009); recombination in the *Foxg1-Cre* line is highly background-dependent (Hebert and McConnell, 2000); and, as *Tgfb3* itself is expressed and required in several other tissues besides MEE and adjacent periderm during early embryogenesis, the *Tgfb3-Cre* knock-in line is of very limited use in studies of palatal epithelial fusion (Yang et al., 2008). As expression of *Tgfb3* in MEE and in periderm is so crucial to normal palatogenesis, occurs in precisely the regions where we would like to regulate the expression of other genes genetically, and its regulation poorly understood, we set out to identify the enhancer sequences responsible for this highly specific expression of *Tgfb3*. Using BAC deletion analysis we were able to identify a 61-kb region around the *Tgfb3* gene that could drive *lacZ* reporter expression specifically in the MEE and adjacent periderm. Expression of this reporter was much more specific to the MEE/periderm than that of the endogenous *Tgfb3* gene, and as this 61-kb region did not drive detectable expression before E12.5 it is a good candidate region for the development of novel palatal epithelium/periderm-specific Cre-driver lines.

In order to use enhancer sequence information to learn more about molecular regulation of *Tgfb3* expression we needed to identify the important sequences more precisely. Palatogenesis is an evolutionarily conserved developmental process in amniote animals (Bush and Jiang, 2012). Mammals and reptiles have a fused secondary palate, and although avians develop a beak and have a naturally cleft palate (Ferguson, 1988) fusion can be induced by exposing the appropriate stage avian palatal shelves (which do not express endogenous *cTgfb3*) to human recombinant TGF-β3 (Sun et al., 1998). Although it was therefore likely that enhancers directing palatal expression would be amongst non-coding highly conserved sequences amongst mammals, these are very numerous, and those that lay within the Tgfb3 gene (ECRI-IV) turned out not to be palatal enhancers. The FaceBase project (www.facebase.org -A. Visel) to identify craniofacial transcriptional enhancers using ChIP-Seq (IP using anti-p300) recently released a dataset obtained on E14 whole palates but this was not helpful for our specific project; within the 61-kb region only a region around *Tgfb3* exon 1 was flagged as being a putative enhancer; we could demonstrate only a putative vascular enhancer in the area of ECR-VII (Figures 3, 4 and data not shown) where the anti-p300 binding was above the background level in ECR-VII (Figure 4).

By directing our analysis outside the *Tgfb3* gene within the 61 kb fragment, we were able to identify a distal 3.5-kb region in *Ift43* intron 2 and a proximal 5.3-kb region encompassing *Ift43* intron 1 and most of the intergenic sequence between the *Ift43* and *Tgfb3* genes able to target the reporter activity to the MEE and adjacent periderm. However, these smaller regions directed less specific and weaker reporter activity than the 61 kb fragment. While we were able to break the distal 3.5-kb region down further into two smaller modules, which again showed further reduced activity, our attempts to narrow down the 5.3-kb proximal region into even shorter sequences were not successful, suggesting that palate-specific reporter activity seen in the larger region was dependent on two or more regulatory elements separately located in the smaller fragments. Although the same approach has yielded relatively short enhancers that drive very precise and strong expression in other cases (Dodou et al., 2004; Chandler et al., 2007), it is established that not all physically concise and robust expression is regulated in such a simple manner (Evans et al., 2012). Genetic regulatory network studies in Drosophila first introduced the concept of “shadow” enhancers (Lagha et al., 2012). Perry et al. reported that, in addition to the proximal primary enhancer located just upstream of the promoter, the *snail* gene is regulated by a distal enhancer located within the neighboring locus (Perry et al., 2010), which they suggested be defined as a “shadow” enhancer. Subsequent studies have suggested that secondary enhancers are needed to obtain sufficient phenotypic robustness to drive tightly controlled expression of important developmental genes (Frankel et al., 2010). Our findings of putative proximal (primary) enhancer(s) and two (or more) distal enhancers in the neighboring upstream gene that work precisely but only weakly in isolation are reminiscent of this mechanism. A “lack of simplicity” may also extend to the organization of enhancers for other tissues and repressive elements controlling *Tgfb3* expression as we noticed that, unlike the 61-kb region which directed reporter activity specifically in the MEE/periderm, many of the smaller domains around the ECR-V also targeted the reporter activity to the vasculature including the palatal vessels. Similar vascular patterns were seen in embryos carrying either ECR-VI or ECR-VII (which were unable to direct expression in the MEE/periderm) suggesting that all three ECRs located in intron 2 of *Ift43* possess putative redundant vascular enhancer activities.

Very little is known about the molecular mechanisms regulating Tgfb3 expression in the epithelial tips of pre-fusion palatal shelves. Venza et al. recently reported that in Foxe1 mutant embryos Tgfb3 expression is dramatically reduced in the palatal epithelium, and that Tgfb3 is a direct target of FoxE1 via FoxE1 binding sites in the Tgfb3 promoter region (Venza et al., 2011). As outlined above, He et al. reported that epithelium-specific mouse mutants lacking the gene encoding β-catenin also show a dramatic reduction inTgfb3 expression in the palatal epithelium suggesting that canonical Wnt signaling is involved in Tgfb3 regulation (He et al., 2011). Whether these identified transcriptional regulators function purely by contributing to the core promoter activity or by also regulating Tgfb3 expression via distal enhancers is not yet known. Nevertheless, even the smallest cis-regulatory region identified in this study (i.e., the 300-bp ECR-V located in the Ift43 intron 2) contained three evolutionarily conserved TCF/LEF consensus binding sites and two FoxE1 binding sites (data not shown) implying that these factors may have the capacity to regulate Tgfb3 in palatal shelf tissues by binding directly to the putative enhancer elements. Thus our results are consistent with existing molecular regulation data, and suggest a model in which the MEE/periderm-specific Tgfb3 expression is achieved via a complex regulatory landscape composed of a putative proximal (primary) enhancer(s) and two (or more) distal shadow enhancers i.e., some that lie in the neighboring upstream gene.

### Conflict of interest statement

The authors declare that the research was conducted in the absence of any commercial or financial relationships that could be construed as a potential conflict of interest.

## References

[B1] Ahmed S. Liu C. C. Nawshad A. (2007). Mechanisms of palatal epithelial seam disintegration by transforming growth factor (TGF) beta3. Dev. Biol. 309, 193–207 10.1016/j.ydbio.2007.06.018 17698055 PMC2084085

[B2] Andl T. Ahn K. Kairo A. Chu E. Y. Wine-Lee L. Reddy S. T. (2004). Epithelial Bmpr1a regulates differentiation and proliferation in postnatal hair follicles and is essential for tooth development. Development 131, 2257–2268 10.1242/dev.01125 15102710

[B3] Behringer R. Nagy A. Gertsenstein M. Vintersten K. (2003). Manipulating the Mouse Embryo - A Laboratory Manual. New York, NY: Academic Press

[B4] Bush J. O. Jiang R. (2012). Palatogenesis: morphogenetic and molecular mechanisms of secondary palate development. Development 139, 231–243 10.1242/dev.067082 22186724 PMC3243091

[B5] Carinci F. Scapoli L. Palmieri A. Zollino I. Pezzetti F. (2007). Human genetic factors in nonsyndromic cleft lip and palate: an update. Int. J. Pediatr. Otorhinolaryngol. 71, 1509–1519 10.1016/j.ijporl.2007.06.007 17606301

[B6] Chandler R. L. Chandler K. J. McFarland K. A. Mortlock D. P. (2007). Bmp2 transcription in osteoblast progenitors is regulated by a distant 3′ enhancer located 156.3 kilobases from the promoter. Mol. Cell. Biol. 27, 2934–2951 10.1128/MCB.01609-06 17283059 PMC1899916

[B7] Dodou E. Verzi M. P. Anderson J. P. Xu S. M. Black B. L. (2004). Mef2c is a direct transcriptional target of ISL1 and GATA factors in the anterior heart field during mouse embryonic development. Development 131, 3931–3942 10.1242/dev.01256 15253934

[B8] Doetschman T. Georgieva T. Li H. Reed T. D. Grisham C. Friel J. (2012). Generation of mice with a conditional allele for the transforming growth factor beta3 gene. Genesis 50, 59–66 10.1002/dvg.20789 22223248 PMC3850393

[B9] Dudas M. Kim J. Li W. Y. Nagy A. Larsson J. Karlsson S. (2006). Epithelial and ectomesenchymal role of the type I TGF-beta receptor ALK5 during facial morphogenesis and palatal fusion. Dev. Biol. 296, 298–314 10.1016/j.ydbio.2006.05.030 16806156 PMC1557652

[B10] Evans N. C. Swanson C. I. Barolo S. (2012). Sparkling insights into enhancer structure, function, and evolution. Curr. Top. Dev. Biol. 98, 97–120 10.1016/B978-0-12-386499-4.00004-5 22305160

[B11] Ferguson M. W. (1988). Palate development. Development 103(Suppl.), 41–60 3074914 10.1242/dev.103.Supplement.41

[B12] Fitzpatrick D. R. Denhez F. Kondaiah P. Akhurst R. J. (1990). Differential expression of TGF beta isoforms in murine palatogenesis. Development 109, 585–595 2401212 10.1242/dev.109.3.585

[B13] Frankel N. Davis G. K. Vargas D. Wang S. Payre F. Stern D. L. (2010). Phenotypic robustness conferred by apparently redundant transcriptional enhancers. Nature 466, 490–493 10.1038/nature09158 20512118 PMC2909378

[B14] Griswold S. L. Sajja K. C. Jang C. W. Behringer R. R. (2011). Generation and characterization of iUBC-KikGR photoconvertible transgenic mice for live time-lapse imaging during development. Genesis 49, 591–598 10.1002/dvg.20718 21309067 PMC3409694

[B15] Gritli-Linde A. (2007). Molecular control of secondary palate development. Dev. Biol. 301, 309–326 10.1016/j.ydbio.2006.07.042 16942766

[B17] He F. Xiong W. Wang Y. Li L. Liu C. Yamagami T. (2011). Epithelial Wnt/beta-catenin signaling regulates palatal shelf fusion through regulation of Tgfbeta3 expression. Dev. Biol. 350, 511–519 10.1016/j.ydbio.2010.12.021 21185284 PMC3040240

[B16] Hebert J. M. McConnell S. K. (2000). Targeting of cre to the Foxg1 (BF-1) locus mediates loxP recombination in the telencephalon and other developing head structures. Dev. Biol. 222, 296–306 10.1006/dbio.2000.9732 10837119

[B18] Iwata J. Suzuki A. Pelikan R. C. Ho T. V. Sanchez-Lara P. A. Urata M. (2013). Smad4-Irf6 genetic interaction and TGFbeta-mediated IRF6 signaling cascade are crucial for palatal fusion in mice. Development 140, 1220–1230 10.1242/dev.089615 23406900 PMC3585659

[B19] Kaartinen V. Cui X. M. Heisterkamp N. Groffen J. Shuler C. F. (1997). Transforming growth factor-beta3 regulates transdifferentiation of medial edge epithelium during palatal fusion and associated degradation of the basement membrane. Dev. Dyn. 209, 255–260 9215640 10.1002/(SICI)1097-0177(199707)209:3<255::AID-AJA1>3.0.CO;2-H

[B20] Kaartinen V. Voncken J. W. Shuler C. Warburton D. Bu D. Heisterkamp N. (1995). Abnormal lung development and cleft palate in mice lacking TGF-beta 3 indicates defects of epithelial-mesenchymal interaction. Nat. Genet. 11, 415–421 10.1038/ng1295-415 7493022

[B21] Lagha M. Bothma J. P. Levine M. (2012). Mechanisms of transcriptional precision in animal development. Trends Genet. 28, 409–416 10.1016/j.tig.2012.03.006 22513408 PMC4257495

[B22] Lidral A. C. Romitti P. A. Basart A. M. Doetschman T. Leysens N. J. Daack-Hirsch S. (1998). Association of MSX1 and TGFB3 with nonsyndromic clefting in humans. Am. J. Hum. Genet. 63, 557–568 10.1086/301956 9683588 PMC1377298

[B23] Millan F. A. Denhez F. Kondaiah P. Akhurst R. J. (1991). Embryonic gene expression patterns of TGF beta 1, beta 2 and beta 3 suggest different developmental functions in vivo. Development 111, 131–143 1707784 10.1242/dev.111.1.131

[B24] Pelton R. W. Dickinson M. E. Moses H. L. Hogan B. L. (1990). *In situ* hybridization analysis of TGF beta 3 RNA expression during mouse development: comparative studies with TGF beta 1 and beta 2. Development 110, 609–620 1723948 10.1242/dev.110.2.609

[B25] Perry M. W. Boettiger A. N. Bothma J. P. Levine M. (2010). Shadow enhancers foster robustness of Drosophila gastrulation. Curr. Biol. 20, 1562–1567 10.1016/j.cub.2010.07.043 20797865 PMC4257487

[B26] Proetzel G. Pawlowski S. A. Wiles M. V. Yin M. Boivin G. P. Howles P. N. (1995). Transforming growth factor-beta 3 is required for secondary palate fusion. Nat. Genet. 11, 409–414 7493021 10.1038/ng1295-409 PMC3855390

[B27] Rajewsky K. Gu H. Kuhn R. Betz U. A. Muller W. Roes J. (1996). Conditional gene targeting. J. Clin. Invest. 98, 600–603 10.1172/JCI118828 8698848 PMC507466

[B28] Rienhoff H. Y. Jr. Yeo C. Y. Morissette R. Khrebtukova I. Melnick J. Luo S. (2013). A mutation in TGFB3 associated with a syndrome of low muscle mass, growth retardation, distal arthrogryposis and clinical features overlapping with Marfan and Loeys-Dietz syndrome. Am. J. Med. Genet. A 161A, 2040–2046 10.1002/ajmg.a.36056 23824657 PMC3885154

[B29] Sun D. Vanderburg C. R. Odierna G. S. Hay E. D. (1998). TGFbeta3 promotes transformation of chicken palate medial edge epithelium to mesenchyme *in vitro*. Development 125, 95–105 9389667 10.1242/dev.125.1.95

[B30] Taya Y. O'kane S. Ferguson M. W. (1999). Pathogenesis of cleft palate in TGF-beta 3 knockout mice. Development 126, 3869–3879 10433915 10.1242/dev.126.17.3869

[B31] Venza I. Visalli M. Parrillo L. De Felice M. Teti D. Venza M. (2011). MSX1 and TGF-beta3 are novel target genes functionally regulated by FOXE1. Hum. Mol. Genet. 20, 1016–1025 10.1093/hmg/ddq547 21177256

[B32] Warming S. Costantino N. Court D. L. Jenkins N. A. Copeland N. G. (2005). Simple and highly efficient BAC recombineering using galK selection. Nucleic Acids Res. 33, e36 10.1093/nar/gni035 15731329 PMC549575

[B33] Wu C. Endo M. Yang B. H. Radecki M. A. Davis P. F. Zoltick P. W. (2013). Intra-amniotic transient transduction of the periderm with a viral vector encoding TGFbeta3 prevents cleft palate in Tgfbeta3(-/-) mouse embryos. Mol. Ther. 21, 8–17 10.1038/mt.2012.135 23089732 PMC3538297

[B34] Xiong W. He F. Morikawa Y. Yu X. Zhang Z. Lan Y. (2009). Hand2 is required in the epithelium for palatogenesis in mice. Dev. Biol. 330, 131–141 10.1016/j.ydbio.2009.03.021 19341725 PMC2745957

[B35] Xu X. Han J. Ito Y. Bringas P. Jr. Urata M. M. Chai Y. (2006). Cell autonomous requirement for Tgfbr2 in the disappearance of medial edge epithelium during palatal fusion. Dev. Biol. 297, 238–248 10.1016/j.ydbio.2006.05.014 16780827

[B36] Yahata K. Maeshima K. Sone T. Ando T. Okabe M. Imamoto N. (2007). cHS4 insulator-mediated alleviation of promoter interference during cell-based expression of tandemly associated transgenes. J. Mol. Biol. 374, 580–590 10.1016/j.jmb.2007.09.054 17945255

[B37] Yang L. T. Li W. Y. Kaartinen V. (2008). Tissue-specific expression of Cre recombinase from the Tgfb3 locus. Genesis 46, 112–118 10.1002/dvg.20372 18257072 PMC2430471

[B38] Yoshida M. Shimono Y. Togashi H. Matsuzaki K. Miyoshi J. Mizoguchi A. (2012). Periderm cells covering palatal shelves have tight junctions and their desquamation reduces the polarity of palatal shelf epithelial cells in palatogenesis. Genes Cells 17, 455–472 10.1111/j.1365-2443.2012.01601.x 22571182

